# Teledermatology scale-up frameworks: a structured review and critique

**DOI:** 10.1186/s12913-018-3418-x

**Published:** 2018-08-07

**Authors:** Laticha Elizabeth Marolana Walters, Richard Ernest Scott, Maurice Mars

**Affiliations:** 1 Department of TeleHealth, Nelson R. Mandela School of Medicine, University of KwaZulu-Natal, Durban, South Africa; 2 Council for Scientific and Industrial Research (CSIR), Meraka Institute, Pretoria, South Africa; 3 Department of Community Health Sciences, University of Calgary, Calgary, Canada; 4 NT Consulting - Global e-Health Inc., Calgary, Canada

**Keywords:** eHealth, Telemedicine, Teledermatology, Scale-up requirements, Scale-up framework, KwaZulu-Natal, South Africa, Developing countries

## Abstract

**Background:**

The South African public health sector embarked on a National Telemedicine System implementation program in 1999 and although unsuccessful, the Province of KwaZulu-Natal subsequently implemented teledermatology in 2003, with two currently active services (synchronous and asynchronous). Although sustained these have not been scaled-up to meet the needs of all hospitals in the Province. A recent teledermatology scale-up design requirements elicitation process within KwaZulu-Natal confirmed the need for a framework, and identified requirements through key stakeholders, programme observations, the literature, and experts. This study aimed to identify and characterise existing teledermatology or related eHealth scale-up frameworks, determine whether any met the previously elicited scale-up framework requirements, and were suitable for use in the KwaZulu-Natal public health sector.

**Methods:**

A structured literature search was performed of electronic databases (Scopus, Science Direct, IEEE, PubMed, and Google Scholar) seeking proposed or developed teledermatology or related scale-up frameworks. Global public health publications were also hand-searched. The teledermatology or telemedicine, telehealth or eHealth related scale-up frameworks identified were critiqued against the previously elicited teledermatology scale-up framework requirements to determine their suitability for use.

**Results:**

No specific teledermatology scale-up framework was found. Seven related scale-up frameworks were identified, although none met all the previously identified teledermatology scale-up framework requirements. The identified frameworks were designed for specific scale-up phases and lacked a more holistic and comprehensive approach.

**Conclusions:**

There is an evidenced-based need for the development of a health sector aligned, holistic framework that meets the identified teledermatology scale-up framework requirements. The findings of this paper will inform development of such a framework.

**Electronic supplementary material:**

The online version of this article (10.1186/s12913-018-3418-x) contains supplementary material, which is available to authorized users.

## Background

It is internationally recognised that virtual access to healthcare can be enabled through the application of various modes of “*information and communication technologies (ICT) for health*” (eHealth) [1]. This includes store-and-forward (asynchronous) telemedicine, real-time interactive (synchronous) telemedicine, or a hybrid technique that combines asynchronous and synchronous communication techniques [2].

A number of diseases initially present with a skin lesion before becoming fully developed [3]. The capacity to effectively and efficiently diagnose and treat dermatological conditions at the district hospital level is important [4]. The visual nature of dermatology lends itself well to the use of eHealth for diagnosis, treatment and monitoring of skin conditions, termed teledermatology (TD). Furthermore there is growing acceptance of diagnostic and treatment concordance provided by TD [5].

Technology applications like TD have important advantages of direct relevance to the developing world by providing ways of mitigating the shortage and maldistribution of specialists, and offering more equitable access to these services by increasing virtual access at the point of care [6]. However, there are few TD initiatives globally that have been successfully and sustainably brought to scale and embedded in routine practice.

In South Africa (SA) innovative solutions and systems are required to address the quadruple burden of disease [7], and supplement the shortage of healthcare workers at all levels [8, 9]. Recognising this, the SA government has identified the need for eHealth (which includes telemedicine and TD) within the National eHealth Strategy [10], mHealth Strategy [11], and draft Telemedicine Strategy [12].

A National Telemedicine System was initiated in SA in 1999, but only phase I of three planned phases of the programme was implemented, with limited success [13] and subsequent phases were not pursued. Failure was attributed to low system utilisation, cost-effectiveness concerns, and technical, organisational and governance challenges [14, 15]. TD was not part of phase I of the National Telemedicine System.

The University of KwaZulu-Natal (UKZN) capitalised on the National Telemedicine System’s investment in ICT and videoconferencing infrastructure by using it for tele-education from 2001 [9], followed by synchronous TD from 2003 onwards [16]. With the use of TD “*52 of the 69 patients (75.4%) were saved a 240 km round-trip to see the specialist dermatologist*” [16: p70c]. In addition, there have been unsuccessful attempts to scale-up the synchronous TD service [17]. A survey reported that TD was one of the most common, all be it limited, implementations of telemedicine in SA [18]. A spontaneous asynchronous TD service has evolved in KwaZulu-Natal (KZN) using smart phones for tele-consultation, whereby a photo is taken, a brief history attached, with both sent via instant messaging services or email [19, 20]. The National Telemedicine System programme, synchronous and spontaneous asynchronous mobile TD, and scale-up attempts provide valuable lessons and requirements for the scaling process.

Despite growth of TD there is no evidence that services have been successfully scaled-up into routine healthcare practices in developing countries such as SA [17]. Scale-up has been defined in several ways: *“the ambition or process of expanding the coverage of health interventions”* [21], *“the process of reaching more people with a proven practice, more quickly, and more effectively in a particular context”* ([22]: p1), and *“deliberate efforts to increase the impact of health service innovations locally tested in pilot or experimental projects, so as to benefit more people and to foster policy and programme development on a lasting basis”* ([23]: p180). This study adopted the latter definition as it relates to a defined need to increase the impact of the two TD services: a successfully tested and long-running synchronous service in three rural public hospitals, and an unplanned spontaneous mobile asynchronous service [20, 24] which could be scaled up to benefit the 67 of 72 provincial public hospitals ([25]: p35, [26]) without a dermatologist. This could be extended and applied to the SA government hospital sector in general.

A study was conducted between 2014 and 2016 based on the current state and future directions of TD in the province of KZN. The study was based on literature review [27], site visits, observations, and semi-structured interviews with key stakeholders such as referring clinicians, consulting dermatologists, and ICT and provincial management [28]. This led to the formulation of the requirements for a teledermatology scale-up framework for KZN [28].

The aim of this study was to identify and characterise existing TD, telemedicine or eHealth related scale-up frameworks and to determine if and how well they met the previously identified TD requirements. The results will be used to determine the need to adopt or adapt any existing framework, or develop a TD scale-up framework (TDSF) for the province of KZN in SA, which may also be applicable in other developing world settings.

## Methods

A structured literature search was used to determine the existence of TDSFs and related scale-up frameworks for eHealth, mHealth, telehealth, or telemedicine, including toolkits and models. These were critiqued to determine if, and to what extent, they met the previously determined TD scale-up requirements.

Five electronic databases: Scopus, Science Direct, IEEE, PubMed and Google Scholar were searched using the following search terms; eHealth, mHealth, telehealth, telemedicine, teledermatology, mobile dermatology, and mdermatology each linked with framework or toolkit, or model and scale or scaling. Databases were searched for publications in English and publication date prior to 2016.

In addition, hand searching of related work was undertaken by reviewing websites of global health bodies and programmes: Advanced Development for Africa (ADA), MEASURE Evaluation PRH (population and reproductive health), MOMENTUM, PATH, World Health Organization (WHO), ExpandNet, and the Pan American Health Organization (PAHO WHO).

Inclusion criteria were that the paper or publication proposed or developed a TD or telemedicine or eHealth related scale-up framework. All authors reviewed the abstracts of identified records and the decision for inclusion was by consensus. Full papers of included resources were obtained for review.

Information extracted from identified frameworks included the framework name; research methods used; theoretical foundations guiding framework design and construction; components that described the building blocks. Considerations and relationships relating to legal, strategy, implementation, operational, the cohesion of components, and real-world implementation or testing of the frameworks were also included.

The key components of identified frameworks were then mapped against three phases: pre scale-up, scale-up and post scale-up. Frameworks were scored for fully or partially meeting the components of a TD requirement.

## Results

**Fig. 1:**
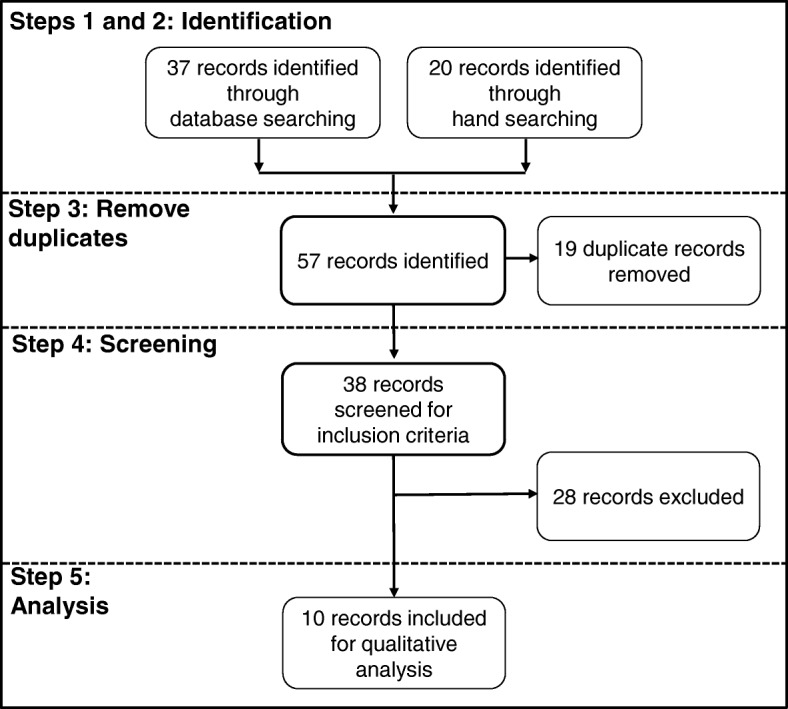
An outline of the search process and results

The search identified 57 records from database and hand searching. After removal of duplicates, a further 28 records were excluded as they did not develop or propose scale-up frameworks, but were reports and discussions of various approaches and assessments. Ten records met the inclusion criteria and were analysed (Fig. 1).

No TD specific scale-up frameworks were identified. Seven related scale-up frameworks were identified in the 10 records (Table 1).

**Table 1:** List of identified frameworks (Abbreviation, Title, Classification and Objective)

Abbreviation	Title of study/ report	Classification	Objective
MAPS	The MAPS toolkit mHealth assessment and planning for scale	mHealth	“to increase the scale of impact of existing mHealth products”([35]: piv)
mHA	Applying a framework for assessing the health system challenges to scaling up mHealth in South Africa	mHealth	“to appraise the opportunities and challenges to effective implementation of mHealth at scale in health systems”([36]: p2)
MTT	Deliverable 3.4 personalised blueprint for telemedicine deployment: Validated and tested version	telemedicine	“main focus is on the 18 critical success factors for telemedicine deployment defined by MOMENTUM. Telemedicine doers need to bear these factors in mind when scaling up their services and deploying them into routine care.”([37]: pii)
SF	Scaling up global health interventions: A proposed framework for success	health interventions	“aimed at planners of scale-up processes to use in thinking about strategies for implementing a new program, policy, or intervention to scale”([29]: p1)
SUF	Practical guidance for scaling up health service innovations	health interventions	“to facilitate the strategic planning and management of the scaling-up process”([30]: p7)
SUM	Scaling up - from vision to large-scale change: A management framework for practitioners	health interventions	“practical advice on a three-step, ten task process for effective scaling up”([34]: p1)
THD	Healthcare without walls: A framework for delivering telehealth at scale	telehealth	“to inform the future NHS strategy for telehealth”([38]: p5)

Three were based on health interventions: framework for success (SF) [29], scale-up framework (SUF) [30–32], and management framework (SUM) [33, 34]; two focussed on mHealth: mHealth toolkit (MAPS) [35] and mHealth assessment framework (mHA) [36]; one on telemedicine: blueprint for telemedicine (MTT) [37]; and one on telehealth: recommendations for telehealth (THD) [38].

### Characterisation of the identified frameworks

The details of the characterisation of the identified frameworks are provided elsewhere (see Additional file 1).

#### Research methods

Qualitative research methods were used in all but one framework [37] which used mixed methods. In addition research approaches varied from implementation research [35], applied research [34], and action research [30]. The most common data collection instruments were literature reviews (6) [29, 30, 34–36, 38], interviews (4) [29, 35, 36, 38], workshops (2) [35, 38], and site visits (2) [35, 36] followed by consultations [35], review panel [35], pre-testing [35], online engagement [38], survey [37], and a user and implementation-driven experience approach [37].

#### Theoretical foundations

There were 10 references to seven frameworks. Diffusion of innovation theory was incorporated in two frameworks – SF [29] and SUF [30]. Others used strategic management (planning, change management, operations) – SUM [34], strategic planning – SUF [30], and social network theory – SF [29]. Practical experience guided the remaining four – MAPS [35], MTT [37], SF [29], and SUF [30]. In addition, reliance was placed on existing models, frameworks and typologies such as Kaiser Permanente’s “*pyramid of care*” model and risk sharing outcomes based payment models - THD ([38]: p20). Three framework approaches (prerequisites for sustainable ICT development, ICT in low middle income countries, and eReadiness assessment) were adopted by mHA [36], and typologies for scaling up considerations SF [29].

#### Components

The main components of frameworks were key categories, processes, domains, dimensions or elements, with sub-components such as sections, levels, focus areas and tasks. Sub-components were supported with strategic choices or questions to guide the scale-up process. In addition three frameworks (SF [29], THD [38], and MTT [37]) developed critical success factors and SUF [30] developed attributes of success as additional mechanisms to support scale-up.

#### Considerations and relationships

THD focused on recommendations for government, the NHS and industry [38]. These formed an action plan for each stakeholder group with critical success factors. mHA adapted three approaches and was based on questions that guided the decision making process on health systems challenges in terms of readiness of government stewardship, and systems on the financial, technical and organisational levels [36]. SF provided implementation success factors made up of opportunities and challenges to consider with critical success factors emphasising learning through action and feedback [29]. SUM’s implementation was guided by a three step process and tasks representing actions and questions on scale-up development and implementation supported with reference tools [34]. The MAPS used requirements for scale-up, partnerships, finance, technology and architecture, operational, monitoring and evaluation areas split into domains that were supported with assessment and planning sections [35]. MTT required that core requirements be met prior to scale-up, based on critical success factors and supporting indicators together with a self-assessment tool [37]. SUF used an open-systems perspective where scaling up was linked with the context in which the innovation was implemented. The design and implementation was driven by seeking “*balance among the elements of the framework*” and supported key attributes to enhance success ([30]: p4).

#### Real-world implementation

Most frameworks were tested for functionality with the intended users, with SUF being recommended for all WHO scale-up programmes [30]. The MAPS [35] and MTT [37] were retrospectively applied to projects of their respective workshop participants and SUM [34] was field tested. The mHA was applied to community-based health data collection and dissemination programmes [36]. THD and SF were developed solely to inform policy planners and makers and there was no evidence of implementation [29, 38].

The detailed results of the TD requirements mapping to identified frameworks are shown elsewhere (see Additional file 2). The identified TD scale-up requirements were mapped against key components of the seven scale-up frameworks. The mapping was done only at a main component level and excluded the detailed activities and reference tools.

The seven identified related scale-up frameworks met many of the requirements to varying degrees, with some scoring three and below (Table 2). In the pre-scale-up phase, two frameworks met the eHealth category ‘Information systems’ together with the TD requirements ‘Architecture’ (MAPS and THD), ‘Information security’ (MAPS and MTT) and ‘User interfaces’ (MAPS and THD). All frameworks partially met the eHealth category ‘Strategy’ together with the TD requirement ‘Clinic setting’. Two frameworks met the eHealth category ‘Benefits’ together with the TD requirement ‘Benefits realisation’ (MTT and THD). None of the frameworks met the eHealth category ‘Scale-up’ and TD requirement ‘Finalisation and close-out’ in the scale-up phase. Only three frameworks met the eHealth category ‘Program management’ together with the TD requirement ‘Risk management’ (MTT, SUF and THD), or the eHealth category ‘Scale-up’ together with the TD requirement ‘Readiness’ (mHA, MTT and THD). Only MAPS and THD met more than 80% of the total requirements.

**Table 2:** Results of mapping key components of identified scale-up frameworks against TD scale-up requirements

eHealth categories	TD requirements	MAPS	mHA	MTT	SF	SUF	SUM	THD
Total and % of TD scale-up framework requirements met (26)		21	12	20	7	17	16	21
		81%	*46%*	*77%*	*27%*	*65%*	*62%*	*81%*
***Pre scale-up phase*** *requirements (17) and % met*		16	*8*	*13*	*4*	*11*	*11*	*15*
		94%	*47%*	*76%*	*24%*	*65%*	*65%*	*88%*
Need	Scale-up need	✓	✓	✓	✓	✓	✓	✓
Stakeholders	Stakeholder management	✓	✓	✓	✓	✓	✓	✓
Strategy	Scale-up strategy and budget	✓	✓	✓	✓	✓	✓	✓
	Environmental scan	✓	✓	✓	✓	✓	✓	✓
	Change management	✓		✓		✓	✓	✓
	Clinic setting	*Partial*	*Partial*	*Partial*	*Partial*	*Partial*	*Partial*	*Partial*
	Availability	✓	✓	✓				✓
Regulations	Regulations	✓		✓		✓	✓	✓
	Standards	✓		✓		✓	✓	✓
Governance	Alignment	✓	✓			✓	✓	✓
Information systems	Architecture	✓						✓
	Information security	✓		✓				
	Automation	✓	✓	✓				✓
	User interfaces	✓						✓
Sustainability	Sustainability	✓	✓	✓		✓	✓	✓
	Operational plan and budget	✓		✓		✓	✓	✓
	Incentives	✓		✓		✓	✓	✓
***Scale-up phase*** *requirements (8) and %*		*4*	*3*	*7*	2	5	4	6
		*50%*	*38%*	*88%*	*25%*	*63%*	*50%*	75%
Benefits	Benefits realisation		*Partial*	✓		*Partial*		✓
	Benefits communication	✓		✓		✓	✓	✓
Program management	Risk management			✓		✓		✓
Scale-up	Mobilisation	✓		✓		✓	✓	
	Readiness		✓	✓				✓
	Training plan	✓	✓	✓	✓	✓	✓	✓
	Support plan	✓	✓	✓	✓	✓	✓	✓
	Finalisation and close-out							
***Post scale-up phase*** *and requirements (1) % met*		*1*	*1*	*0*	*1*	*1*	*1*	*0*
		*100%*	*100%*	*0%*	*100%*	*100%*	*100%*	*0%*
Monitoring	Monitor and control	✓	✓	*Partial*	✓	✓	✓	*Partial*

## Discussion

The study did not identify an existing TDSF. Seven related scale-up frameworks with a focus on health interventions, telehealth and telemedicine were found. No framework met all the requirements of the previously identified three scale-up phases: pre scale-up, scale-up, and post scale-up. Two frameworks (MAPS and THD) met more than 80% of the pre scale-up requirements, none met all the scale-up phase requirements, and most (five) met all the post scale-up phase requirements.

The lack of an existing TDSF could potentially be ascribed to TD services growing by learning through action, experience, and using lessons learned in an iterative cycle. Botswana shared their TD scale-up journey with sustainability criteria, but did not propose a framework [39]. The most practical advice identified was the success factors shared by the United Kingdom from their TD integration into routine healthcare, but again no formal framework was proposed [40]. The results of mapping TD scale-up requirements against components of related scale-up frameworks indicated that the highest scores stem from research approaches based on informed inputs “*for doers, by doers*” based on user and implementation experience ([37]: p1), active key stakeholder engagements [38], and implementation [35], action [30], and applied research [34]. Furthermore Hanson (2010) confirms that scale-up is to be supported with “*learn through action*” and incorporate “*lessons of experience*” to develop and refine scale-up strategies and implementation plans ([41]: p3).

The frameworks that met most of the requirements were originally developed to scale-up telehealth and mHealth and were developed by global health or non-governmental organisations. There is a paucity of implementation details in real-world settings, although most had been validated in some form. An exception was the THD [38] where no evidence of validation or implementation was reported. The frameworks comprised mainly of components and sub-components, with tools to guide the scale-up decision-making and planning processes. Four frameworks (SF [29], SUF [30], THD [38], and MTT [37]) had critical success factors as additional mechanisms to enhance and monitor scale-up success.

The overall relationships or organisation of the framework components and considerations were assessed to determine the intention of the framework to support scale-up [42]. According to Simon (1996),*“Resemblance in behavior of systems without identity of the inner systems is particularly feasible if the aspects in which we are interested arise out of the organization of the parts, independently of all but a few properties of the individual components”* ([42]: p17). The SUF used an open-systems perspective where scaling-up was linked with the context in which the innovation was implemented and continuously adjusted to “*balance*” the elements ([30]: p45). The MAPS [35] was based on an iterative process from assessment, planning and improvements; THD [38] extended the framework to an action plan per stakeholder group. This perspective is well aligned to Simon (1996) who stated that the extent to which the inner environment (such as the TD scale-up requirements) met the needs of the outer environment (which in the local context is the Department of Health in KZN), then the artefact (namely the TDSF) will meet its intended objectives [42]. This balance was strengthened with critical success factors and practice guidelines. Some frameworks have not elaborated on how the organisation of their components can ensure realisation of scale-up objectives, but relied more on considerations and reference tools to guide the process.

The lack of identified frameworks to meet the TD scale-up requirements for all scale-up phases could reflect a focus of the authors to address a particular scale-up phase; this presents an opportunity for more comprehensive and holistic development needs. Despite the fact that frameworks recognised the benefits of eHealth, the active identification and linking to indicators through benefits realisation management was not elevated to the key component level. Unless scale-up objectives are linked to tangible public health benefits and actively managed from the onset, scarce resources could potentially be wasted. In addition frameworks generally lacked specific focus on planning activities for ‘information systems’, e.g., architecture, information security, and user interfaces [28]. Similarly scale-up implementation activities lacked risk management under ‘Program management’. Risk management cannot be assumed but has been identified as a key ingredient for implementation success [43].

Van Dyk (2014) and van Gemert-Pijen et al. (2011) confirmed the need for more holistic framework development [44, 45]. Scaling-up of a health intervention cannot happen in a vacuum, but requires a multifaceted [40] and holistic approach [44, 45]. Successful scaling may require a holistic approach whereby all scale-up phases are adequately addressed. Within SA and the KZN setting there is a need for such an integrated approach to embrace telemedicine in general and TD in particular.

The ExpandNet [31, 32] and MTT [37] approaches state that planning for scale needs to be intentional and made from the onset of a programme. In addition, planning needs to be comprehensive and across all the phases of a scale-up process to ensure all the requirements are adequately addressed.

Yamey refers to the relative advantage gained from understanding the association between meeting the needs of the adopter (in this case the referring doctors, dermatologists and the public health system), and the resultant faster diffusion of the innovation (in this case the scale-up of TD) [29]. The diffusion of innovation theory supports the view that interventions are to be needs-based [46] and that the results will reflect how well these have been met. Within KZN, the scale-up of TD has the inherent capability to meet the needs of the public sector on a number of levels, such as increasing access to specialist dermatologists, enhancing the effectiveness and efficiency of the referral system, supporting continuing professional development [9, 47], and increased job satisfaction for referring clinicians (physician and nurse) [48, 49].

A scale-up framework provides a systematic guide to planning and implementation processes, leading to sustained practice [31]. Furthermore, the use of a scale-up framework can guide the systematic recording and feedback of lessons learned to enhance scaling success and sustainability. Due to the nature of the eHealth categories (strategy, governance, information systems, sustainability, benefits, program management, scale-up, and monitoring) in Table 2, the authors considered COBIT 5 [50] an IT governance and management framework, but excluded it due to the “perceived complexity” ([51]: p403).

A combination of the strengths of MAPS and THD could be considered to inform the development of an evidenced-based TDSF for KZN with the addition of TD requirements under the pre scale-up phase (clinic setting, information security), and scale-up phase (benefits realisation management, risk management, readiness and finalisation and close-out).

The review is limited to the search terms, inclusion criteria and availability of literature prior to 2016. Furthermore identified frameworks were characterised and critiqued against teledermatology scale-up framework requirements defined for the KwaZulu-Natal Department of Health in South Africa [28]. Also requirements mapping was limited to the main components of the identified frameworks. Future work could consider expanding the search terms to include variants of scale or scaling but will need to be verified against the scale-up definition. In addition the proposed approach of Walters et al. (2018) can be used to define context specific scale-up framework requirements to inform framework characterisation and critique [28]. The potential results from using variants of scale-up could possibly identify additional related scale-up frameworks, although the results of a recent study [52], despite using wider search terms, are consistent with the need for holistic framework development, whilst the availability of a teledermatology scale-up framework (TDSF) will not be affected.

## Conclusions

There is no TDSF available and none of the related scale-up frameworks identified met all the TD scale-up requirements for the KwaZulu-Natal Department of Health. There is therefore a need to develop a health sector aligned, holistic, well balanced TDSF that meets the identified TD requirements and economic pressures, whilst enabling learning through incorporating strengths of identified frameworks, experience and feedback in an iterative cycle.

## Additional files

Additional file 1: Details of characterisation of the identified frameworks. Detailed listing of characterisation of identified frameworks split into Abbreviation, Research methods, Theoretical foundations, Components, Considerations and relationships, Real-world implementation; (DOCX 240 kb)

Additional file 2: Detailed results of the TD requirements mapping to identified frameworks. Detailed listing of the results of the TD requirements mapping to the components of the identified frameworks (DOCX 248 kb)

## Abbreviations

HIV/AIDS: Human immunodeficiency virus infection and acquired immunodeficiency syndrome
KZN: KwaZulu-Natal
SA: South Africa
TD: Teledermatology
TDSF: Teledermatology scale-up framework
UKZN: University of KwaZulu-Natal
MAPS: Toolkit mHealth assessment and planning for scale
mHA: Framework for assessing the health system challenges to scaling up mHealth
MTT: Blueprint for telemedicine deployment
SF: Proposed framework for success
SUF: Practical guidance for scaling up health service innovations
SUM: Management framework for practitioners
THD: Framework for delivering telehealth at scale
CSIR: Council for Scientific and Industrial Research
ICT: Information communication technology
