# Supplementary material for: Teledermatology scale-up frameworks: a structured review and critique
Source: BMC Health Serv Res. 2018 Aug 7;18:613. doi: 10.1186/s12913-018-3418-x (PMC6081905; doi:10.1186/s12913-018-3418-x)
Supplement: Supplementary file 1 — Details of characterisation of the identified frameworks. Detailed listing of characterisation of identified frameworks split into Abbreviation, Research methods, Theoretical foundations, Components, Considerations and relationships, Real-world implementation; (DOCX 240 kb) [file 12913_2018_3418_MOESM1_ESM.docx]

Additional file 1: Details of characterisation of the identified frameworks (Abbreviation, Research methods, Theoretical foundations, Components, Considerations and relationships, Real-world implementation)

| Framework abbreviation | Research methods | Theoretical foundations (keywords = theory/ model/ framework/ experience) | Components | Considerations and relationships | Real-world implementation |
| --- | --- | --- | --- | --- | --- |
| MAPS [1] | Implementation research: literature review, workshops,  site visits, interviews, consultations,  review panel, pre-testing | Scale-up experienced-based (challenges and lessons learned) | **Components**: Six “axes of scale” that are split into sixteen domains are defined with three sections: thematic overview, self-assessment questions and planning and guidance activities with reference materials.   1. **Groundwork:** Parameters of scale, contextual environment, scientific basis 2. **Partnerships:** Strategic engagement, partnership sustainability 3. **Financial health:** Financial management, financial model 4. **Technology & architecture:** Data, interoperability, adaptability 5. **Operations:** Personnel, training and support, outreach and sensitisation, contingency planning 6. **Monitoring & evaluation:** Process monitoring, evaluation research | **Phases (keywords = phase/ steps/ sequencing)** “scaling up as a continuous process and offers insights that will prove useful throughout the on-going stages of mHealth project goals for scaling up and sustainability, whether projects are focused on government adoption, commercial adoption, or a hybrid model” p1  **Considerations:** Scale up requirements, health policy, partnerships, finance, technology and architecture, operational, monitoring and evaluation  **Relationships:** An “axis” is split into one or more domain and supported with assessment and planning sections. Iterative process from assessment, planning and improvements. | Applied the toolkit to the projects of the network of Innovative Working Group mHealth project grantees during a validation workshop in Malawi. |
| mHA [2] | A qualitative study: literature review, interviews, site visits, | Placed a reliance on the underlying theories of the three adapted approaches | **Components:** Four health system dimensions with nine key elements to be considered:   1. **Government stewardship:** strategic leadership, learning environment; 2. **Organizational systems:** culture of information use, capacity for implementation; 3. **Technological systems:** usability, interoperability, privacy and security; 4. **Financial systems:** sustainable funding, cost effectiveness | **Phases (keywords = phase/ steps/ sequencing)** no details provided  **Considerations:** Using a framework adapted from three approaches: sustainable ICT development in LMIC, infrastructural and cultural context and systems challenges, and eReadiness assessment.  **Relationships:** Every dimension a question and have specific elements that need to be considered in decision making | Community-based health promotion and prevention, and health awareness education |
| MTT [3] | Survey, user and implementation experience | No specific details provided but tools used: practical experience, Model for Assessment of Telemedicine (MAST), Common Working Model (CALLIOPE project) | **Components:** Two core prerequisites followed by four domains and four levels/ elements with 18 critical success factors (CSF) and 51 indicators for telemedicine implementation  **Core Prerequisites:** IT system design and evidenced based intervention -   1. **Context:** Critical success factor (CSF) - Cultural readiness, compelling need 2. **People:** CSF - Leadership, stakeholder involvement, patient centeredness and user friendliness 3. **Plan:** CSF - Resource aggregation, primary client, business plan, change management, legal and security conditions, potential for scale-up 4. **Run:** CSF - Legal and security guidelines, legal and security experts, privacy awareness, IT and eHealth infrastructure, service monitoring and market procurement   **Levels/ elements:** Strategy and management, Organization and management, Legal and security, Technology and market | **Phases (keywords = phase/ steps/ sequencing) phases are mentioned such as** scale-up phase, test phase, pilot phase, transition phase, various phases of implementation are mentioned but it does not form part of the framework and dependencies. Stages are used but more “stages of a pilot process” p33 or “They also indicated that the toolkit might be used more than once at different  stages of telemedicine deployment” p33. The timetable refers to some stages but it’s not integral to the framework.  **Considerations:** **Critical success factors (18)** and supporting indicators (51)  **Relationships:** Two core prerequisites must be met, followed by the critical success factors and supporting indicators. Tools and techniques included to guide self-assessment process | Validated with UNITED4Health project in Kristiansand, Norway |
| SF [4] | Literature review, interviews | Adapted two typologies: constraints to scaling, and elements of scaling up.  Diffusion and social network theories and implementation experience | **Components:** Six success factor attribute categories and 13 success factors   1. **Specific tool or service being scaled up:** simplicity, scientifically robust technical policies 2. **Implementers:** strong leadership and governance, engaging local implementers and other stakeholders, using both state and non-state actors as implementers 3. **Chosen delivery strategy:** applying diffusion and social network theories, cascade and phased approaches to scale-up, tailoring scale-up to the local situation and decentralizing delivery, adopting and integrated approach to scale-up 4. **‘‘Adopting’’ community:** an engaged “activated” community 5. **Socio-political context:** political will and national policies, country ownership 6. **Research context:** incorporating research into implementation | **Phases (keywords = phase/ steps/ sequencing) List phases under “delivery strategy” “**A related concept is the notion of going to scale in a phased manner, beginning with a pilot program, followed by stepwise expansion, learning lessons along the way to help refine further expansion” p2 - this is a secondary reference from “Quelapio MID, Mira NRC, Orillaza-Chi RB, Belen V, Mun˜ez N, et al. (2010) Responding to the multidrug-resistant tuberculosis crisis: mainstreaming programmatic management to the Philippine National Tuberculosis Programme. Int J Tuberc Lung Dis 14: 751–757.”  **Considerations:** Implementation success factors (13)**,** opportunities and challenges  **Relationships:** Strategies to consider when implementing and critical success factors.  Emphasis on learning by doing and feedback into strategies for implementation | Developed to inform policy planners and makers |
| SUF [5] | Strategic assessment, action research: literature review, experience and evidence-based | Strategic planning, diffusion of innovation and through practical experience | **Components:** Five elements with scaling-up strategy as the main element, supported with strategic choices and recommendations with a nine step process and four key principles   1. The innovation 2. User organisation 3. Environment 4. Resource team 5. Scaling-up strategy: Type of scaling up - dissemination and advocacy, organisational process, costs/ resource mobilisation, monitoring and evaluation | **Phases (keywords = phase/ steps/ sequencing)** strategic choice area where decisions on the type of scaling-up is selected i.e. the pace to be phased or gradual rapid. “Gradual, phased expansion of the innovation is often needed for successful scaling up” p37 [5]  Nine step process with pace selection to the type scaling  **Considerations:** Policy, organization, social sciences, technology transfer, diffusion of innovation, and research utilization approach  **Relationships:** An open-systems perspective where scaling up is linked with the context in which the innovation is implemented.  The design and implementation is driven by seeking “balance among the elements of the framework” supported with case information from the field.  Attributes of success propose pointers for implementation. | Recommended for all WHO scale up programmes |
| SUM [8, 9] | Applied research: literature review, experience and field feedback | Strategic management (planning, change management, operations) | **Components:** Three step process, ten tasks and questions   1. **Develop a scaling up plan:** Create a vision, assess scalability, fill information gaps, prepare a scaling up plan 2. **Establish the pre-conditions for scaling up:** Legitimize change, build a constituency, realign and mobilise resources 3. **Implement the scaling up process:** Modify organisational structures, coordinate action, track performance and maintain momentum | **Phases (keywords = phase/ steps/ sequencing)** three step process with ten tasks  **Considerations:** Scale up, strategic planning and organisational developments  **Relationships:** The tasks are recommended actions complemented with questions (developing and implementing scale-up plan) and tools (planning and implementing scale-up strategy) to guide scaling | Field tests in India, Nigeria and Mexico and shared with academic, policy makers and practitioners |
| THD [10] | Literature review, interviews, workshops and online engagement | Incorporated the Kaiser ‘pyramid of care’ model, risk sharing outcomes-based payment models | **Components:** Five sections supported with an action plan for four groups to action: local NHS, commissioned (national), corporate (central), industry and 11 key success factors   1. **Policy context – the need for action:** national policy drivers including the NHS White Paper; 2. **Commissioning and funding:** the evidence; investment, funding and reimbursement options and preferred recommendations; 3. **Care redesign:** how telehealth can be moved beyond ‘pilot-itis’ towards mainstream care provision, including organisational change best practice, the patient experience and stakeholder engagement; 4. **Service delivery:** alternative service models, education and training, legal and ethical considerations; 5. **Enabling scale:** implications for national and local infrastructure (both public and NHS), and standards; integration with core patient record systems, at a national and local level; market readiness. | **Phases (keywords = phase/ steps/ sequencing)** refer to transition phase but no phase or step approach outlined  **Considerations:** Recommendations for government, the NHS and industry more broadly four groups for action**:**  Local NHS, commissioned (national), corporate (central), industry  **Key success factors (11):** clinical leadership, locally and nationally; projects as a change initiative; project roll-out at the local level; project thresholds; build on successful pilots; build on where PCTs can offer the change management and leadership capacity; importance of available support, expertise and capability; importance of data quality from a range of data sources; need for greater publicity; effort involved in getting all stakeholders involved; contracting out of the management of patients with specific long term conditions  **Relationships:** Action plan with recommendations to support key issues and focus areas per action group and key success factors | Developed to inform policy planners and makers |
