# Supplementary material for: Teledermatology scale-up frameworks: a structured review and critique
Source: BMC Health Serv Res. 2018 Aug 7;18:613. doi: 10.1186/s12913-018-3418-x (PMC6081905; doi:10.1186/s12913-018-3418-x)
Supplement: Supplementary file 2 — Detailed results of the TD requirements mapping to identified frameworks. Detailed listing of the results of the TD requirements mapping to the components of the identified frameworks (DOCX 248 kb) [file 12913_2018_3418_MOESM2_ESM.docx]

Additional file 2: Detail results of the TD requirements mapping to identified frameworks

| TD requirement | MAPS [1] | mHA [2] | MTT [3] | SF [4] | SUF [5, 6, 7] | SUM [8, 9] | THD [10] |
| --- | --- | --- | --- | --- | --- | --- | --- |
| **TD scale-up need**  *(Evidenced-based health system need that is prioritized, technologically appropriate and culturally sensitive) [11]* | Under domain 3 – Scientific basis – it is required for “project teams to demonstrate:   - that the mHealth product is aligned with scientifically validated health interventions - general evidence of the effect of the mHealth product on identifiable problems; - context-specific evidence supporting the appropriateness of the tool for a certain setting (e.g. the content has been adapted and validated for local use)” p12 | **“**Cost-effectiveness: The cost-effectiveness of mHealth strategies is evaluated. mHealth interventions are weighed up against other priority and evidence-based interventions (in terms of the costs, resources and capacity requirements), and opportunity costs are routinely considered. Unintended consequences of introducing new technology within a weak health system are monitored to minimize negative effects.” P6 | Evidenced based intervention | **“**Simmons and Shiffman argue  that successful scale-up ‘‘requires the  systematic use of evidence to guide the  process and incorporate new learning’’ p3 although evidence-based need is not seen as a requisite but using research evidence to inform the scaling process. | “Plan on being cautious about initiating scale-up before the required evidence is Available” p9 [7] | Taking the time to do an evaluation, assessment, and  comparison with alternatives is important  and, ideally, should be done by someone  who is detached and independent” p18 | Under the section Business case and commissioning it is highlighted that “On the back of WSD results and other relevant evidence, prepare a full cost-benefit analysis around the national potential from scaling up telehealth” p8 |
| **Change management**  *(Consideration for TD’s impact on existing routine operations: practice guidelines, changes in clinical and administrative workflow, workload, and staffing structures) [11]* | Change is considered under financial management and “health system cost” and workflow although not referred to as change management | No specific consideration | Change management | No specific consideration | The term change used throughout | Change management, business process reengineering | Change management |
| **Clinic setting**  *(physical TD room considerations for patient privacy, confidentiality, equipment, and layout) [11]* | No specific but general consideration: referred to physical infrastructure **“**When deploying mobile-based communications systems in low-resource settings, projects will inevitably encounter technical barriers in the forms of lack of connectivity, physical infrastructure and unstable power sources” p72 | No specific but general consideration: referred more to policy and ICT environment | No specific but general consideration: | No specific but general consideration: environmental characteristics and environment of the change are considered | No specific but general consideration: implementation capacity | No specific but general consideration: social, external environments are considered | No specific but general consideration: clinical governance and patient considerations |
| **Availability**  *(TD at nearest point of care) [11]* | Availability considered | Point of care consideration | Availability consideration | No specific consideration | No specific consideration more reference to policy and political support availability | No specific consideration | Availability considered |
| **Alignment**  *(TD aligned to eHealth governance) [11]* | Consideration for partnership sustainability | Strategic leadership ensures alignment | Not a key consideration. Consideration for alignment towards the service but no scale-up alignment to overall ehealth governance | Governance mentioned but no consideration of scale-up alignment to ehealth governance | “Alignment with policy and programme priorities” p12 (2009) | **“**In cases where the values and norms of the adopting and originating organizations are dissimilar, part of the scaling-up effort requires transforming the model or aligning theses values so that transfer can occur” P39 | “If good governance is already in place within NHS organisations, these practices should form the basis of any contract with private providers” p57 |
| **Architecture**  *(TD information technology architectural plan to leverage on eHealth architecture) [11]* | Considered | No specific consideration | No specific consideration for architecture but infrastructure was considered | No specific consideration | No specific consideration | No specific consideration | “there is a need to build an architecture that supports that diversity, allowing acute, community, mental health and GP systems to exchange information,  and the Coalition Government’s commitment to continuing the Summary Care Record becomes important in this context” p72 |
| **Information security**  *(comply with legislation and best practices) [11]* | Considered | No specific consideration | “A legal risk assessment is a process that runs parallel to an information security risk Assessment” p20 | No specific consideration | No specific consideration | No specific consideration | No specific consideration |
| **Automation systems**  *(electronic health information systems) [11]* | Considered | Considered | Considered | No specific consideration | No specific consideration | No specific consideration | Considered |
| **User interface**  *(user-friendly to enable data accuracy and completeness) [11]* | Considered | No specific consideration | No specific consideration | No specific consideration | No specific consideration | No specific consideration | Considered the different types of interfaces |
| **Sustainability**  *(alignment to universal health coverage) [11]* | Considered | Considered | Considered | No specific consideration | Considered | Considered | Considered |
| **Operational plan and budget**  *(planning for sustained scale-up funding) [11]* | Considered | No specific consideration | Considered | No specific consideration | Considered | Considered | Considered |
| **Incentives**  *(attract and retain dermatologists) [11]* | Considered | No specific consideration | Considered | No specific consideration | Considered | Considered | Considered |
| **Benefits realisation**  *(TD benefits realisation management approach) [11]* | No specific consideration | Benefits were identified although uncertainty as to how it can be sustained | Consideration for cost benefit analysis and measuring patient benefits | No specific consideration | Consideration for cost versus benefits although benefits realisation management not specific | No specific consideration | value of clinic and economic benefits identified and recommended for management |
| **Benefits communication**  *(TD contribution to ehealth indicators) [11]* | Considered | No specific consideration | Patient benefit indicators | No specific consideration | Dissemination and advocacy | Impacts of scaling, tangible milestones, strategic communications | Considered |
| **Risk management**  *(identify, communicate and manage scale-up risks) [11]* | Some data security but not scale-up risks | No specific consideration | Legal, security and implementation risks considered | No specific consideration | Considered | No specific consideration | Considered |
| **Mobilisation**  *(confirm planned resources are approved) [11]* | Considered | No specific consideration | Considered | No specific consideration | Considered | Considered | No specific consideration |
| **Readiness**  *(cut-over from implementation to operational scaled-up state) [11]* | No specific consideration | Considered | Considered | No specific consideration | No specific consideration | No specific consideration | Considered |
| **Training plan**  (end-user clinical, administrative and project team training) [11] | Considered | Considered | Considered | Considered | Considered | Considered | Considered |
| **Support plan**  *(support during and post scale-up) [11]* | Considered | Considered | Considered | Considered | Considered | Considered | Considered |
| **Finalisation and close-out** *(account for scale-up deliverables and resource transitioning) [11]* | No specific consideration | No specific consideration | No specific consideration | No specific consideration | No specific consideration | No specific consideration | No specific consideration |
| **Monitor and control** *(progress of planned versus actual and corrective actions during planning, implementation and after scale-up) [11]* | Used process monitoring and make “course corrections” pvii. Axes of scale of monitoring and evaluation. | Used terms monitoring and evaluation – although control element not considered. | **“**monitor the service” p31 does not relate to monitor during and after scale-up process and “The form of service monitoring explored here is technological in character” p26. Cost control are considered although not process controls to ensure continued performance and conformance | **“**The active participation of the community in planning, implementing, and monitoring interventions is widely cited as a crucial factor in successful scale-up” p3 “Incorporating research into implementation  (‘‘learning and doing’’) p3 – lean on research to provide control/ feedback information | Refer to consideration for on-going monitoring and evaluation and linkages to decision making although control not specifically used | Consideration for monitoring and evaluation “Task 10 covers monitoring and evaluating progress and feeding that information back into public oversight and modification of the model” p38 | Emphasis on telehealth using monitoring devices or technologies and not scale-up monitoring. Control is considered under local care re-design |

**References**

1. World Health Organization: The MAPS Toolkit mHealth Assessment and Planning for Scale. <http://apps.who.int/iris/bitstream/10665/185238/1/9789241509510_eng.pdf> (2015). Accessed 18 November 2017.

2. Leon N, Schneider H, Daviaud E: Applying a framework for assessing the health system challenges to scaling up mHealth in South Africa. *BMC Medical Informatics and Decision Making. (*2012); 12:123. <https://doi.org/10.1186/1472-6947-12-123>.

3. Jensen LK, Knarvik U, Pedersen CD, Tangene W, Whitehouse D: Deliverable 3.4 Personalised Blueprint for telemedicine deployment: validated and tested version version. <http://www.telemedicine-momentum.eu/wp-content/uploads/2015/02/D3.4_v1.0_ValidatedBlueprint.pdf> (2015). Accessed 18 November 2017.

4. Yamey G: Scaling up global health interventions: A proposed framework for success. *PLoS Med. (*2011); 8(6):e1001049. <https://doi.org/10.1371/journal.pmed.1001049>.

5. World Health Organization: Practical guidance for scaling up health service innovations. <http://apps.who.int/iris/bitstream/10665/44180/1/9789241598521_eng.pdf> (2009). Accessed 18 November 2017.

6. World Health Organization: Nine steps for developing a scaling-up strategy. Geneva, Switzerland.: WHO and ExpandNET; (2010). <http://www.who.int/immunization/hpv/deliver/nine_steps_for_developing_a_scalingup_strategy_who_2010.pdf>.

7. World Health Organization: Beginning with the end in mind: planning pilot projects and other programmatic research for successful scaling up. France: WHO; (2011). [http://www.expandnet.net/PDFs/ExpandNet-WHO - Beginning with the end in mind - 2011.pdf](http://www.expandnet.net/PDFs/ExpandNet-WHO%20-%20Beginning%20with%20the%20end%20in%20mind%20-%202011.pdf).

8. Cooley L, Kohl R: Scaling up—From vision to large-scale change: A Management framework for practitioners. <http://www.msiworldwide.com> (2006). Accessed.

9. Cooley L, Rajani RV: Scaling up—From vision to large-scale change: A Management framework for practitioners. <http://www.msiworldwide.com/wp-content/uploads/Scaling-Up-Framework.pdf> (2012). Accessed 21 November 2017.

10. Cruickshank J, Beer G: Healthcare without walls: A framework for delivering telehealth at scale: 2020health.org; (2010). <http://www.2020health.org/2020health/Publications/Publications-2010/telehealth.html>.

11. Walters LEM, Scott RE, Mars M: Design requirements for a Teledermatology Scale-up Framework. In*.*: [Under review]; (2017).
